# CXCL13, CXCL10 and CXCL8 as Indicators of Ocular and Neurological Involvement in Patients With Ocular Syphilis: An Observational Descriptive Study

**DOI:** 10.3389/fopht.2022.916718

**Published:** 2022-06-27

**Authors:** Laurie W. van der Merwe, Candice Snyders, Martin Kidd, Novel N. Chegou, Gerhard Walzl, Derrick P. Smit

**Affiliations:** ^1^ Division of Ophthalmology, Faculty of Medicine and Health Sciences, Stellenbosch University, Cape Town, South Africa; ^2^ Department of Science and Innovation - National Research Foundation (DSI-NRF) Centre of Excellence for Biomedical Tuberculosis Research; South African Medical Research Council Centre for Tuberculosis Research; Division of Molecular Biology and Human Genetics, Faculty of Medicine and Health Sciences, Stellenbosch University, Cape Town, South Africa; ^3^ Centre for Statistical Consultation, Stellenbosch University, Stellenbosch, South Africa

**Keywords:** CXCL13, CXCL10, CXCL8, neurosyphilis, ocular syphilis, uveitis

## Abstract

**Aim:**

To investigate the role of the chemokines CXCL13, CXCL10 and CXCL8 in the diagnosis of ocular- and neurosyphilis by examining the serum, aqueous humour (AH) and cerebrospinal fluid (CSF) of patients with ocular syphilis.

**Methods:**

An observational descriptive study was performed prospectively at Tygerberg Academic Hospital in Cape Town, South Africa from 1 February 2018 till 31 January 2021 which enrolled 23 participants. 14 Patients were male and 9 female, 15 patients were HIV positive, and all patients were newly diagnosed with ocular syphilis. Upon diagnosis of ocular syphilis, the HIV status of each patient was determined, and 3 samples (AH, serum and CSF) were collected to measure the levels of CXCL13, CXCL10 and CXCL8 in each. All patients were treated with 14 days of intravenous Penicillin G and topical corticosteroid drops for uveitis.

**Results:**

The mean concentrations of all 3 biomarkers were higher in the AH and CSF than in the serum. The mean concentrations of the 3 measured biomarkers were markedly different when comparing both AH and CSF levels to serum levels. The level of CXCL13 measured in the AH correlated well with the concentrations found in the CSF of patients with neurosyphilis. In patients with neurosyphilis, mean AH levels of CXCL13 and CXCL10 were markedly higher than in serum while mean CSF levels of CXCL10 were also markedly higher than in serum. Also, the AH/serum ratio of CXCL13 and CXCL10, as well as the CSF/serum ratio of CXCL10, was much higher in patients with neurosyphilis than without. In patients with HIV infection, mean AH CXCL13 levels were much higher than in patients without HIV infection.

**Conclusion:**

The levels of CXCL13, CXCL10 and CXCL8 in the AH of patients with neurosyphilis are similar to previously reported levels in the CSF of patients with neurosyphilis and can potentially be an adjunct in the diagnosis of ocular syphilis. Patients with ocular syphilis who tested negative for neurosyphilis with conventional CSF testing showed features of neurosyphilis when analysing the CSF chemokines.

## Introduction

The incidence of syphilis, caused by the spirochaete *Treponema pallidum*, decreased towards the end of the 20th century when penicillin became available and public health measures improved (1). However, since the start of the 21st century, an increasing number of cases of syphilis and ocular syphilis were recorded (1–8). The Centers for Disease Control and Prevention (CDC) reported a 74% increase in syphilis cases in the United States between 2015 and 2019 (2). The risk factors most frequently documented are male sex, homosexuality and intravenous drug abuse, but the infection rates are also increasing in females and heterosexual individuals (1–8). In 3 studies from South Africa, syphilis was identified as the cause of uveitis in 7% to 16.2% of cases (9–11).Ocular syphilis is diagnosed when ocular inflammation occurs in conjunction with a serologically confirmed systemic syphilis infection, and other causes for inflammation have been excluded. The relationship between ocular syphilis and neurosyphilis has been much debated, but it is assumed that, due to the embryological origin of the retina from the central nervous system, ocular syphilis is a clinical feature of neurosyphilis. Although this is widely accepted for the involvement of the optic nerve and retina, it is disputed whether anterior uveitis represents an entity apart from neurosyphilis (12, 13).

The CDC recommends that a baseline examination of the cerebrospinal fluid (CSF) should be performed for all patients with ocular syphilis, and treatment should commence as for symptomatic neurosyphilis, with 18–24 million units intravenous aqueous crystalline penicillin G per day for 10–14 days, even with a normal CSF examination (14) Alternatives to intravenous aqueous crystalline penicillin G would be intramuscular procaine penicillin G 2.4 million units daily plus Probenecid 500 mg orally four times per day, both for 10 – 14 days. If a patient is allergic to penicillin Ceftriaxone 1 -2 g daily as intravenous or intramuscular injection is advised (14). Ocular syphilis can, nevertheless, manifest as various ocular syndromes, and no differentiation is made regarding the type of ocular involvement.

However, the International Ocular Syphilis Study Group surveyed a group of ophthalmologists, which comprised 103 ophthalmologists from 35 countries, and found that only 40.8% of the participants performed routine CSF examination, 49.5% only tested the CSF if certain clinical findings were present (optic nerve involvement, HIV or other) and 9.7% never examined the CSF (7). Only 10.7% performed PCR tests for *T pallidum* on ocular fluid (7). All participants diagnosed patients with ocular syphilis by serological testing and clinical presentation, and 95% of the participants treated patients with this diagnosis with antibiotics prescribed for 10 days or more, but only 61.8% of participants treated ocular syphilis with intravenous aqueous penicillin G and 24.5% treated with intramuscular benzathine penicillin G (duration not specified) (7).

PCR and immunoblot analysis of ocular fluid have been used to aid in the diagnosis of ocular syphilis, either as a part of the diagnostic workup or in cases where serological results were inconclusive (14–16). In non-ocular syphilis the sensitivity of PCR to detect *T. pallidum* is reported as 72.8% and specificity of 95.5% (17). In a study by Smit et al. none of the 13 participants diagnosed with ocular syphilis tested PCR positive for ocular syphilis on AH sampling, whereas immunoblot assays confirmed syphilis in 3 of the cases (13). Other case series reported variable sensitivity of PCR on ocular fluid for detecting T. pallidum (15, 16). In 1 series, PCR testing of vitreous humor produced positive results even after repeated PCR testing of AH was negative (15). In the other, PCR testing of AH was positive in 3 of 5 cases (16). It appears that the sensitivity of PCR on ocular fluid in ocular syphilis is thus less than that of non-ocular syphilis and it is still uncertain whether vitreous sampling may provide a higher yield than AH (15). The CSF findings of patients with ocular and/or neurosyphilis can be confusing, as 30–70% of patients with neurosyphilis can have a false negative Venereal Disease Research Laboratory (VDRL) test, especially in advanced syphilis (2, 18, 19). Patients co-infected with HIV also pose a unique diagnostic challenge as HIV can cause a CSF pleocytosis (18). VDRL testing of the CSF is seen as the gold standard for diagnosing neurosyphilis, but the high false negative rate has led to the development of additional diagnostic criteria as proposed on the UpToDate (UTD) database, which we used as guidelines for diagnosing neurosyphilis in this study (20).

The chemokine CXCL13 is part of the CXC chemokine family, consisting of 1 amino-acid residue enclosed by the 2 *N*-terminal cysteines, and is also known as B-lymphocyte chemokine 1 (BLC-1) or B-cell attracting chemokine-1 (BCA-1). CXCL13 is secreted by secondary lymphoid tissue, dendritic cells and lymph nodes and is involved in the regulation and migration of B-cells, correlating well with the concentrations of intrathecal B- and T-cells (21–23),and has been investigated as a biomarker for confirmed and asymptomatic neurosyphilis (18, 21–25).

CXCL13 has been found to be upregulated in the CSF of patients with spirochaetal neuroinflammation related to neuroborreliosis and neurosyphilis. This has also been observed in patients with other neuroinflammatory disorders such as multiple sclerosis, cryptococcus infections, CNS lymphoma and asymptomatic HIV infection (18, 23, 25–27). The CXCL13 levels in the CSF of patients with neurosyphilis exceeded 250 pg/ml and was second only to the concentrations found in patients with neuroborreliosis (26).

The aim of our study was to determine if CXCL13, CXCL10 and CXCL8 can be used to facilitate the diagnosis of ocular syphilis by examining the concentrations of these biomarkers in the serum, aqueous humour (AH) and CSF of patients with ocular syphilis and correlating these results with the clinical presentation, HIV status and routine serological and CSF tests, and to assess the association between ocular syphilis and neurosyphilis. To the best of our knowledge, the AH and CSF levels of these chemokines have not previously been measured in patients with ocular syphilis.

## Material and Methods

### Study Participants

An observational descriptive study was performed prospectively at Tygerberg Academic Hospital, Cape Town, South Africa, from 1 February 2018 till 31 January 2021. All patients presenting to the eye clinic with ocular syphilis were recruited if they were older than 18 years, had a positive serum *T. pallidum* antibodies (TPA) result, had an RPR titre of ≥8 and had confirmed ocular inflammation. Patients were excluded from this study if they declined to participate or had another identified cause for the uveitis. Written informed consent was obtained from all participants.

### Investigations

Permission for the study was granted by the Health Research Ethics Committee of the University of Stellenbosch (N13/10/146). Upon diagnosis of ocular syphilis, AH, serum and CSF samples were collected before the initiation of treatment. The HIV status of each patient was ascertained, if they were already on antiretroviral treatment they were also included in the study. All relevant clinical and demographic data were documented, and the patients were treated according to the CDC guidelines. All samples were barcoded and registered on an electronic database before being placed in an automated cryostorage system (Hamilton BIOS, Hamilton Storage GmbH, Bonaduz, Switzerland). Prior to analysis, the samples were removed from cryostorage, thawed, and analysed in a controlled environment. Biomarker concentrations were evaluated using the Luminex^®^XMAP technology platform. The Luminex^®^ platform is a bead-based system and permits simultaneous analysis of several biomarkers in one specimen, based on the principles of flow cytometry. It delivers fast, cost-effective results with up to 500 analytes measurable in one microplate well.

The following biomarkers were measured using the R&D Human Magnetic Luminex Screening Assay kits (R&D Systems Inc, BioTechne, Minneapolis, USA): C-Reactive protein (CRP), CCL4 (MIP-1 beta), CCL24 (Eotaxin-2/MPIF-2), CXCL7 (NAP-2), CXCL10 (IP-10/CRG-2), IFN-gamma, IL-2, IL-6, IL-10, IL-12 p70, CXCL13 and CXCL8. Apo AI and Apo CIII concentrations were assessed using the APOMAG-62K Milliplex Map Human Apolipoprotein Magnetic Bead Panel (Merck Millipore [EMD Millipore] Billerica, MA, USA). For this study the levels of CXCL13, CXCL10 and CXCL8 were measured in the AH, CSF and serum.

### Statistical Analysis

Biomarker levels were compared between sources using mixed model ANOVA. In these analyses the patients were included as a random effect, and sample type, groupings like neurosyphilis (yes/no) were included as fixed effects. Normal probability plots were inspected for deviations from normality, and in suspect cases transformations were done but the results were similar to the untransformed analyses. Thus, only untransformed results were reported. For *post hoc* analyses Fisher least significant difference (LSD) were used.

## Results

### Demographics and Clinical Characteristics

A total of 23 patients with an age range of 22–56 years were included in our study. The most common clinical feature was anterior uveitis (11 patients, 47%), followed by panuveitis, optic neuropathy and intermediate uveitis. CSF features consistent with neurosyphilis were present in 56.5% (13 patients) of the participants, and 65.2% (15 patients) were also infected with HIV. (**Table 1**).

**Table 1:** Demographics and clinical features.

Total	23
**Gender**	
Male	14 (60.9 %)
Female	9 (39.1 %)
**Age in years**	
Median	36
Range	22 – 56
**HIV status**	
Negative	8 (34.8 %)
Positive	15 (65.2 %)
**Eyes affected**	
Unilateral	6 (26.1 %)
Bilateral	17 (73.9 %)
**Clinical features**	
Anterior uveitis	11 (47.8 %)
Panuveitis	8 (34.8 %)
Intermediate uveitis	1 (4.4 %)
Optic neuropathy	3 (13 %)
**Neurosyphilis**	
Yes	13 (56.5 %)
No	10 (43.5 %)

### Results of Biomarkers

#### Ocular Syphilis

The mean concentrations of the 3 biomarkers were measured, and the levels in the eye, central nervous system and blood were compared.

**Figure 1:**
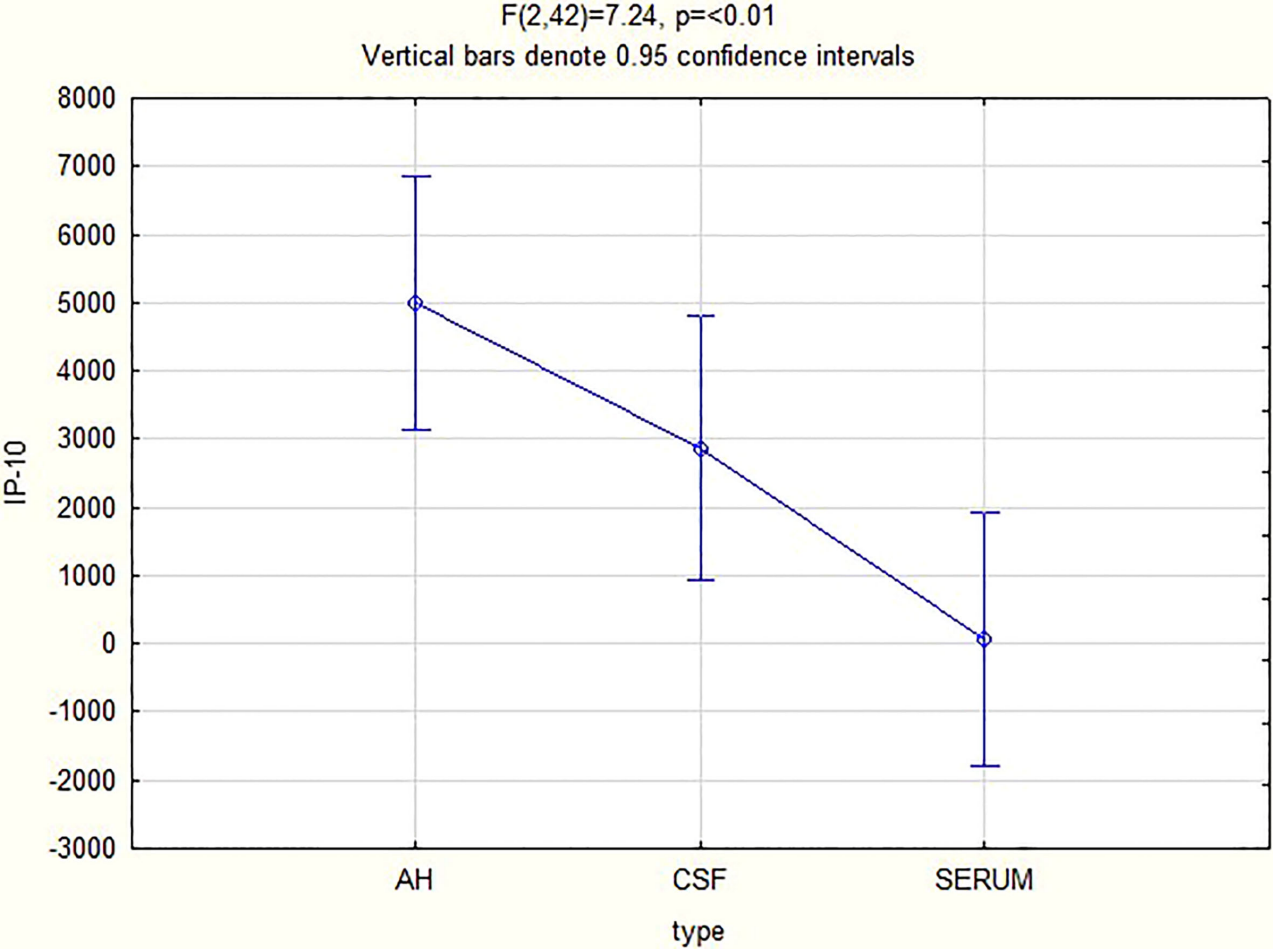
CXCL13 levels in AH, CSF and serum.

**Figure 2:**
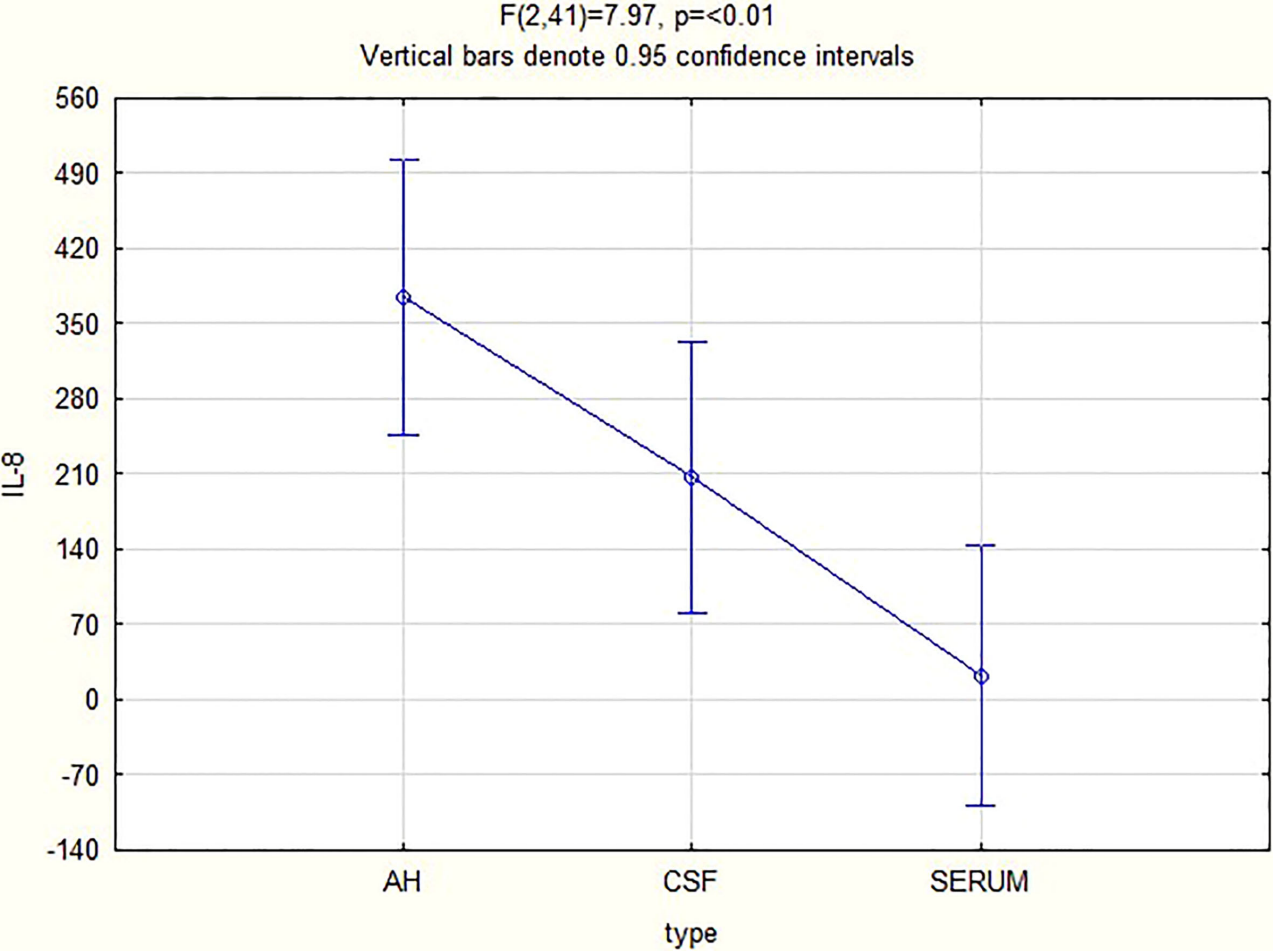
CXCL10 levels in the AC, CSF and serum.

**Figure 3:**
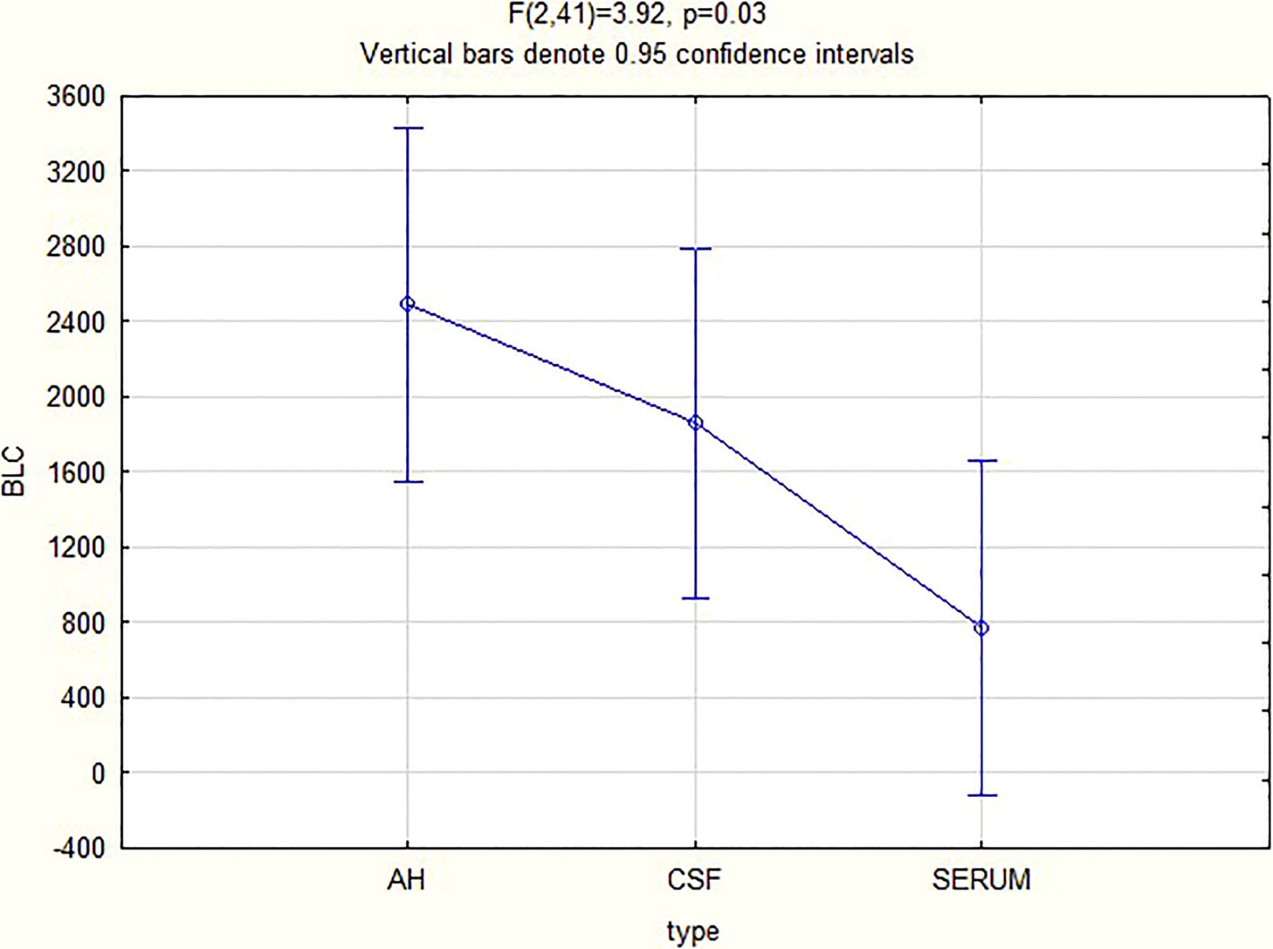
CXCL8 levels in AH, CSF and serum.

For all 3 biomarkers the mean concentrations were higher in the AH and CSF than the serum (**Figures 1**–**3**). Even though the mean concentrations of all 3 chemokines were consistently higher in the AH than the CSF, the differences in mean concentrations between the AH and CSF were insignificant (CXCL13 p=0.39, CXCL10 p=0.06, CXCL8 p=0.13). In contrast, there were marked differences between the mean concentrations of all 3 biomarkers in the AH and serum (CXCL13 p=0.01, CXCL10 p<0.01, CXCL8 p<0.01). When comparing the mean values of all 3 biomarkers in the CSF and serum, there was a noticeable trend for the mean concentrations to be higher in the CSF than the serum (CXCL13 p=0.09, CXCL10 p=0.05, CXCL8 p=0.06).

When considering all patients with ocular syphilis, the overall AH/serum ratio was 3.33 for CXCL13, 84.07 for CXCL10 and 16.89 for CXCL8.

#### Neurosyphilis

Participants were diagnosed with neurosyphilis using CSF test results according to the UpToDate guidelines for patients with and without HIV (20). Different algorithms are used to diagnose neurosyphilis depending on the HIV status of patients:

- A positive CSF VDRL result confirms the diagnosis in both groups.
- In HIV-positive patients:
   o with a non-reactive CSF VDRL, a CSF lymphocyte count of >20 cells/μl suggests a diagnosis of probable neurosyphilis.
   o If the CSF lymphocyte count is between 6–20 cells/μL, but the CD4 count is <200/μL, the patient is receiving antiretroviral (ARV) therapy and the HIV viral load is <50 copies/mL, the patient would be diagnosed with probable neurosyphilis.
   o If any of the preceding criteria are negative, treatment for probable neurosyphilis should be initiated if the CSF fluorescent treponemal antibody-absorption test result is positive (20).
- In patients who tested negative for HIV with a negative VDRL result, a lymphocyte count of >5 cells/μL or CSF protein level >45 mg/dL suggests a diagnosis of probable neurosyphilis (20).

**Figure 4:**
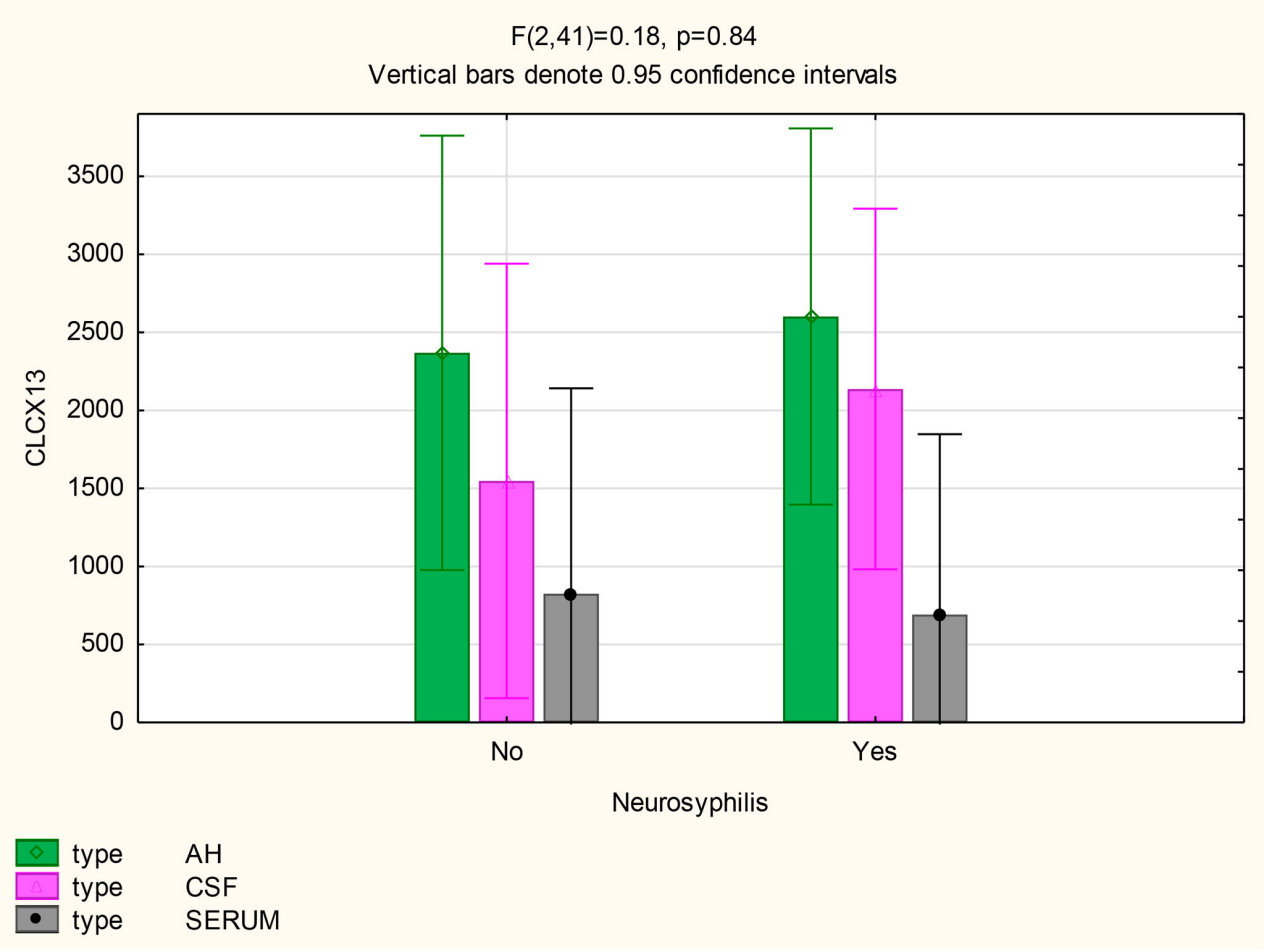
CXCL13 levels in the AH, CSF and serum of patients with and without neurosyphilis.

For participants with and without neurosyphilis, there were no significant differences in the mean concentrations of CXCL13 in the AH (p=0.8), serum (p=0.88) and CSF (p=0.52) (**Figure 4**). However, the mean CXCL13 concentration in the AH was higher than in the serum of participants with neurosyphilis (p=0.02), while there was only a trend for the mean CXCL13 concentration to be higher in the CSF than the serum in these participants (p=0.07) (**Table 2**).

**Table 2:** Actual values of each individual chemokine for each compartment also showing values corrected for neurosyphilis and HIV status.

Average values of each biomarker per compartment						
	AH mean	AH St. Dev.	CSF mean	CSF St. Dev.	Serum mean	Serum St. Dev.
**CXCL13**	2492,4195	2501,5593	1887,6718	2257,1363	749,3622	1176,6273
**CXCL10**	5159,2826	5844,9388	3219,1095	4780,2034	61,3652	45,2839
**CXCL8**	343,4376	468,3988	219,8559	198,8413	20,3304	37,6963
**CXCL13 Neurosyphilis negative**	2328,0733	2494,6549	1527,7956	2402,4421	824,35	1698,9005
**CXCL10 Neurosyphilis negative**	4771,606	5702,163	1410,2644	2283,5701	62,059	48,1132
**CXCL8 Neurosyphilis negative**	480,0356	679,1973	148,0356	180,3777	34,188	55,5042
**CXCL13 Neurosyphilis positive**	2615,6792	2610,1925	2136,8169	2214,3768	691,6792	604,2961
**CXCL10 Neurosyphilis positive**	5457,4954	6166,4536	4471,3869	5688,4812	60,8315	44,9736
**CXCL8 Neurosyphilis positive**	240,9892	191,5481	269,5777	202,3701	9,6708	3,3946
**CXCL13 HIV negative**	1206,0425	1896,0158	1031,39	2006,3316	320,5375	543,3194
**CXCL10 HIV negative**	5518,3363	6199,2453	739,3563	1241,8878	55,5263	52,804
**CXCL8 HIV negative**	347,3688	546,299	131,985	131,5207	30,1713	59,0681
**CXCL13 HIV positive**	3284,0362	2559,4137	2376,9757	2313,718	978,0687	1366,3256
**CXCL10 HIV positive**	4967,7873	5861,4777	4636,1114	5489,5516	64,4793	42,4078
**CXCL8 HIV positive**	341,0185	437,6691	270,0679	216,9977	15,082	20,0908

**Figure 5:**
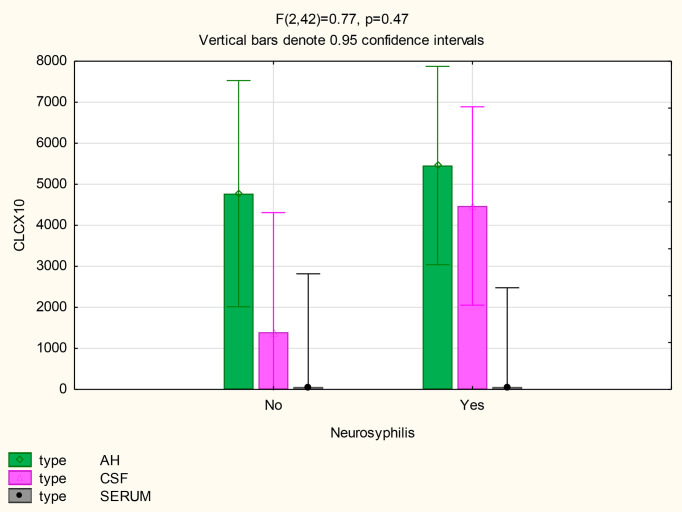
CXCL10 levels in the AH, CSF and serum of patients with and without neurosyphilis.

There were also no differences in the mean concentrations of CXCL10 in the AH (p=0.71), serum (p=1) and CSF (p=0.11) of participants both with and without neurosyphilis. However, in patients with neurosyphilis, the mean CXCL10 concentrations were higher in the AH (p<0.01) and in the CSF (p=0.01) than the serum (**Figure 5** and **Table 2**).

**Figure 6:**
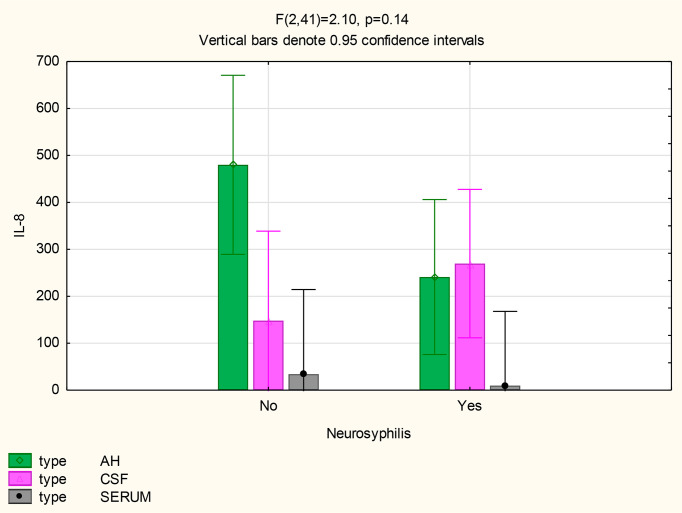
CXCL8 levels in the AH, CSF and serum of patients with and without neurosyphilis.

The mean concentrations of CXCL8 did not differ between the CSF (p=0.33) and serum (p=0.84) of patients with and without neurosyphilis, but the CXCL8 levels trended higher in the AH of participants without neurosyphilis (p=0.06). The mean AH concentrations of CXCL8 in participants without neurosyphilis were also higher than serum concentrations in participants both with and without neurosyphilis (**Figure 6** and **Table 2**).

In patients with ocular syphilis and neurosyphilis the AH/serum ratio for CXCL13, CXCL10 and CXCL8 was 3.78, 89.72 and 24.92 respectively while in patients with ocular syphilis without neurosyphilis the AH/serum ratio for CXCL13, CXCL10 and CXCL8 was 2.82, 76.89 and 14.04 respectively.

#### HIV Infection

**Figure 7:**
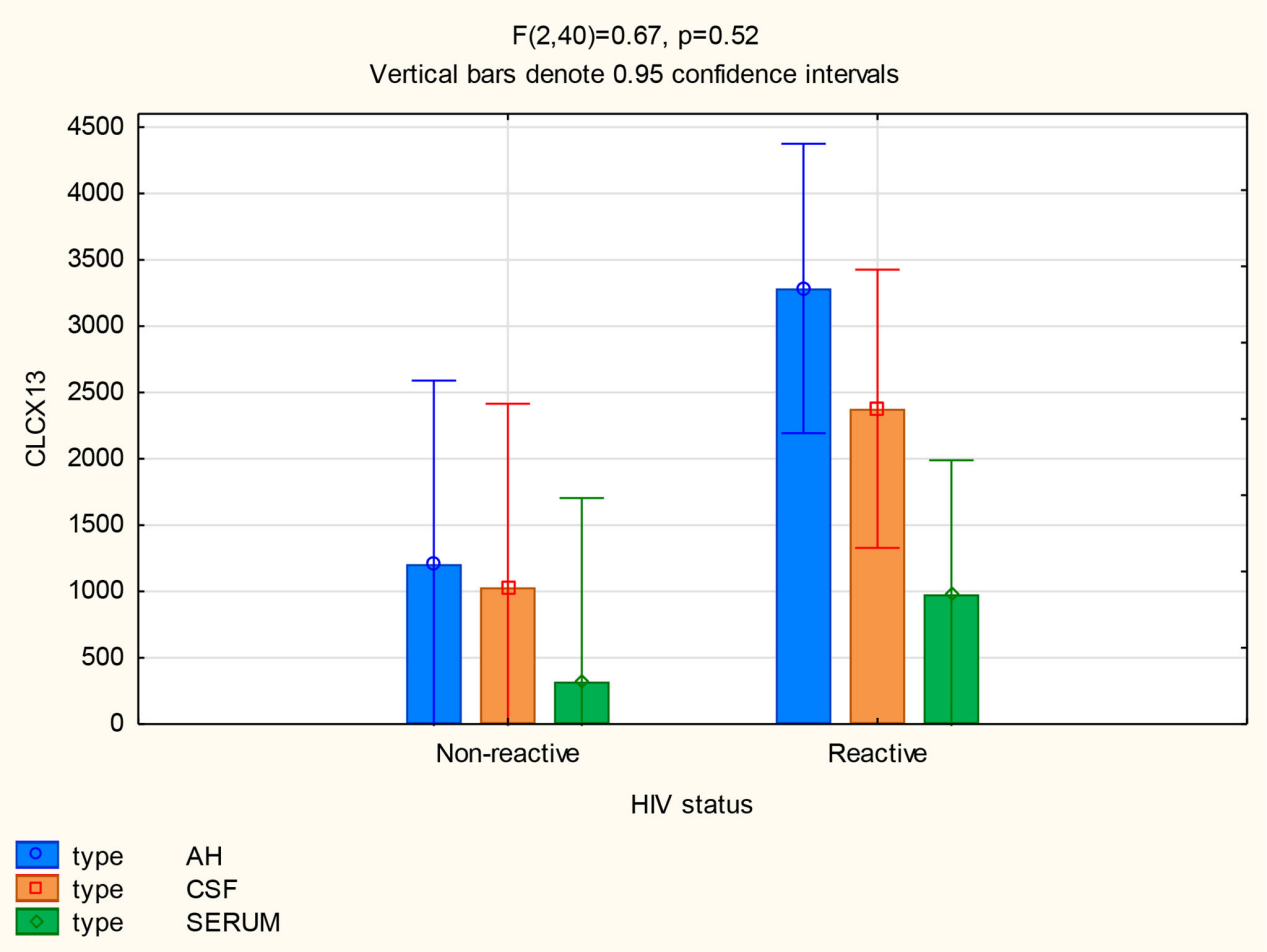
CXCL13 levels in the AH, CSF and serum of patients with and without HIV infection.

There was no significant difference in the mean CXCL13 concentrations in the CSF (p=0.13) and serum (p=0.45) between patients with and without HIV, but the CXCL13 levels were significantly higher in the AH of participants infected with HIV (p=0.02) (**Figure 7**). The mean concentrations of CXCL13 in the AH of participants infected with HIV were also higher than the serum concentrations in participants both with and without neurosyphilis (p<0.01) and the CSF concentrations of participants not infected with HIV (p=0.01) (**Table 2**).

**Figure 8:**
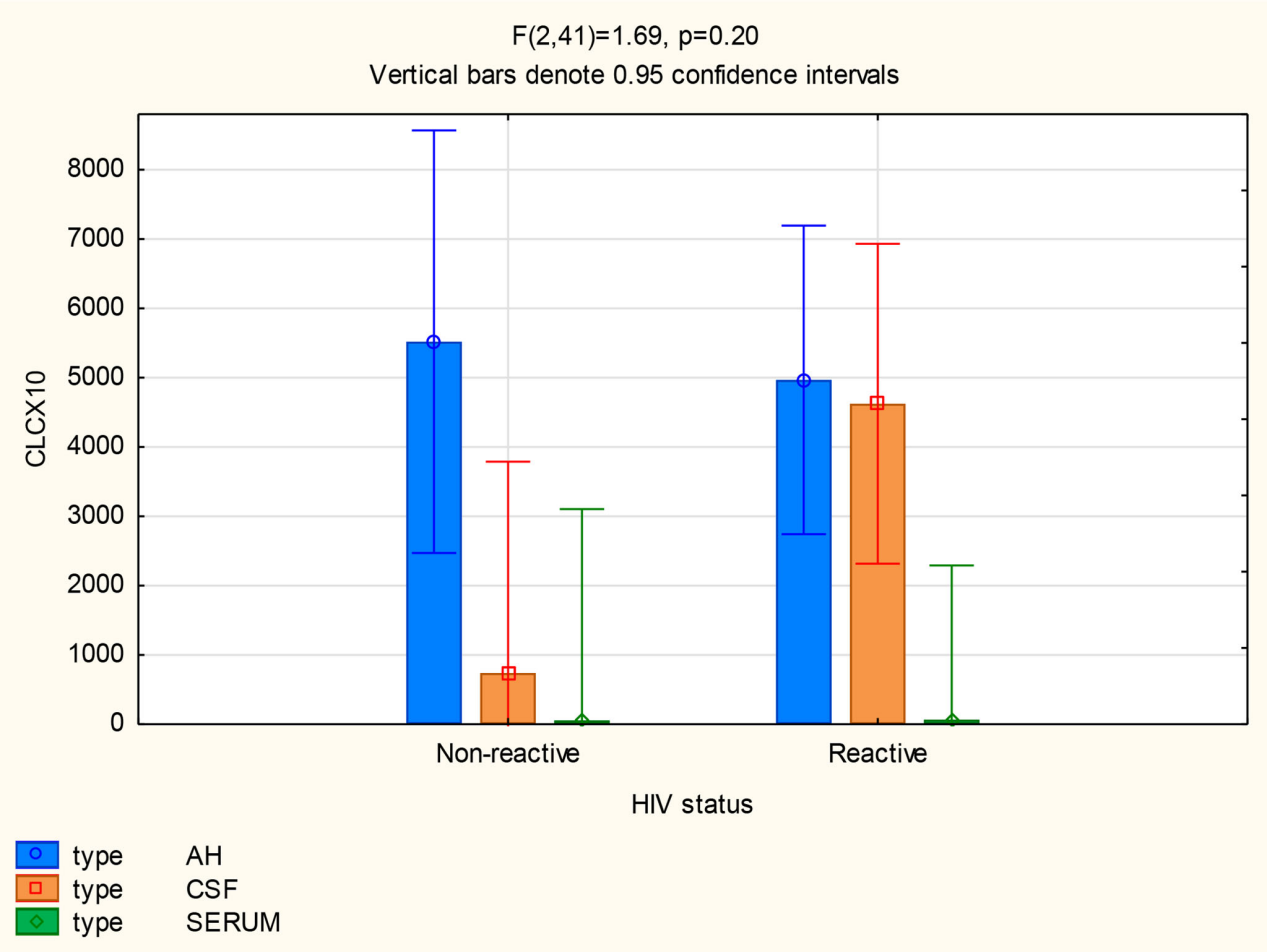
CXCL10 levels in the AH, CSF and serum of patients with and without HIV infection.

The mean concentrations of CXCL10 in the AH (p=0.77) and serum (p=1.00) did not differ between participants with and without HIV, although there was a trend for CXCL10 levels to be higher in the CSF of participants infected with HIV (p=0.05) (**Figure 8**). The mean CSF concentration of CXCL10 was higher than the serum concentration of participants both with (p<0.01) and without (p=0.02) HIV, whereas the mean AH concentrations of CXCL10 in both groups were higher than the serum concentration of participants with (p=0.01) and without (p<0.01) HIV (**Table 2**).

**Figure 9:**
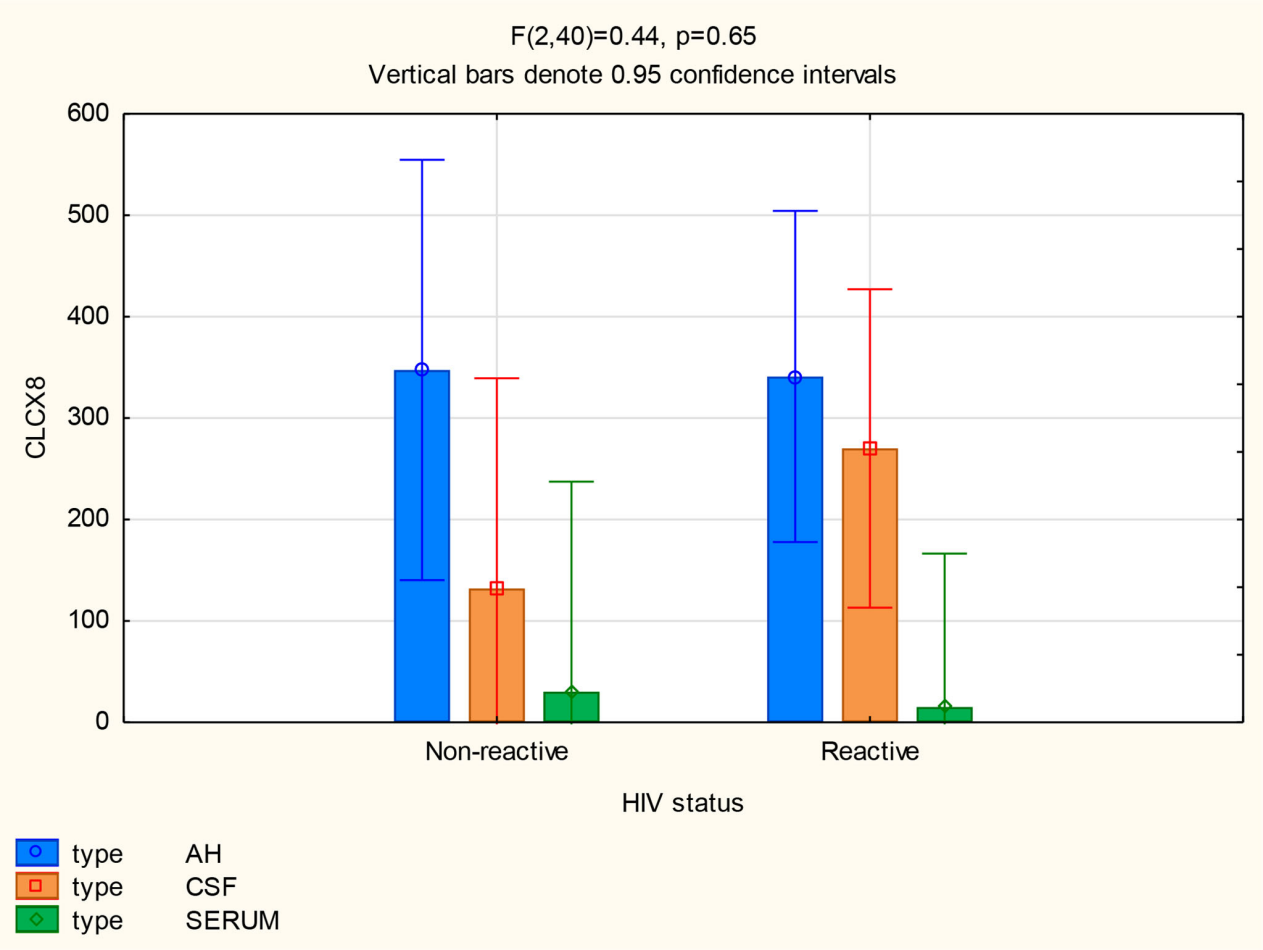
CXCL8 levels in the AH, CSF and serum of patients with and without HIV infection.

The mean concentrations of CXCL8 did not differ between the AH (p=0.96), CSF (p=0.29) or serum (p=0.91) for participants both with and without HIV (**Figure 9**). The mean serum concentrations of CXCL8 in patients with HIV were markedly lower than the AH concentrations of participants with (p<0.01) and without (p=0.01) HIV, and the mean serum concentrations of CXCL8 in HIV negative patients were also lower than the AH concentrations of participants with (p=0.02) and without (p=0.04) HIV (**Table 2**).

#### Anatomical Distribution

Assessment of the mean chemokine levels of patients that presented with anterior uveitis, panuveitis, optic neuropathy and intermediate uveitis (**Table 3**) showed all groups except the patients with optic neuropathy to have significantly elevated levels of chemokines in the AH and CSF, especially when compared to the serum. All 4 groups had mean CSF CXCL13 levels above 250 pg/mL.

**Table 3:** Mean biomarker levels for patient according to clinical phenotype.

Clinical syndrome	Number	Neurosyphilis (UTD*)	HIV infected	Compartment	Mean CXCL13 (pg/mL)	Mean CXCL10 (pg/mL)	Mean CXCL8 (pg/mL)
**Anterior uveitis**	11 (47.8 %)	Yes 5	Yes 6	AH	**1963,4** (61.4 - 5593.4)	**5577,5** (97.9 - 9591.4)	**535,3** (17.8 - 1631.4)
** **		No 6	No 5	CSF	**1452,3** (9.1 - 5303.4)	**1338,8** (47.9 - 6553.4)	**153,5** (31.8 - 517.2)
** **				Serum	**932,2** (41.1 - 5600)	**66,7** (11.0 - 153.2)	**26,0** (3.4 - 173.9)
**Panuveitis**	8 (34.7 %)	Yes 6	Yes 8	AH	**3629,1** (52.4 - 5600)	**5197,1** (268.6 - 13861.1)	**240,8** (0.2 - 658.1)
** **		No 2	No 0	CSF	**2013,5** (11.6 - 5600)	**7036,8** (325.6 - 14200)	**274,9** (45.6 - 718.6)
** **				Serum	**716,8** (255.6 - 1989.9)	**59,2** (18.61 - 148.52)	**19,2** (5.8 - 87.1)
**Optic neuropathy**	3 (13 %)	Yes 2	Yes 0	AH	**12,3** (9.1 - 18.6)	**4746,1** (0.4 - 14187.7)	**28,6** (1.1 - 82.1)
		No 1	No 3	CSF	**1953,2** (16.3 - 5600)	**1497,3** (204.9 - 3692.1)	**199,5** (33.3 - 403.8)
				Serum	**109** (9.1 - 269.3)	**44,3** (13.4 - 92.8)	**3,9** (1.9 - 7.3)
**Intermediate uveitis**	1 (4.3 %)	Yes 1	Yes 1	AH	**5600**	**1496,2**	**382,1**

(*UTD – patients showing CSF findings consistent with neurosyphilis according to UpToDate guidelines for diagnosing neurosyphilis).

On analysis of the sample values of each patient (**Table 4**), 21 CSF samples (95%) had a CSF CXCL13 level of more than 10 pg/mL, and 12 samples (54%) had a CSF CXCL13 level of more than 250 pg/mL. For 11 of the 21 participants (50%) the CXCL13 CSF to serum ratio was more than 1.

**Table 4:** Mean biomarker levels for individual patients.

Summary of CXCL13 levels and clinical details for each patient included in the study													
Ant uveitis	AH	CSF	Serum	Neurosyphilis UTD*	CSF VDRL	CSF FTA Abs	CSF Protein (g/L)	CSF Lymphocytes	CSF CXCL13 > 10 pg/mL	CSF CXCL13 > 250 pg/mL	HIV	CXCL13 CSF:serum	CXCL13 AH:serum
1	5600	11,6	150,75	No	0	Negative	0,14	1	Yes	No	No	0,07	37,14
2	1698,4	9,12	237,61	No	0	Positive	0,28	0	No	No	No	0,04	7,16
3	1078,94	5600	410,3	No	0	Positive	0,41	2	Yes	Yes	Yes	13,6	2,62
4	5593,46	361,24	950,88	No	0	Positive	0,37	3	Yes	Yes	Yes	0,4	5,9
5	1034,98	35,24	162,78	No	0	Positive	0,24	4	Yes	No	No	0,2	6,35
6	363,17	5303,44	350,24	Yes	2	Negative	0,55	34	Yes	Yes	Yes	15,1	1,04
7	Insufficient	2080,26	5600	No	0	Positive	0,34	4	Yes	Yes	Yes	0,4	Insufficient
8	Insufficient	16,3	327,41	Yes	0	Positive	0,55	6	Yes	No	Yes	0,05	Insufficient
9	1023,81	222,19	378,57	Yes	0	Positive	0,67	68	Yes	No	Yes	0,6	2,7
10	61,44	41,1	41,1	No	0	Negative	0,42	0	Yes	No	No	1	1,5
11	1216,72	2294,59	1644,99	Yes	Discontinued	Positive	0,45	6	Yes	Yes	No	1,4	0,7
Mean	1963,4	1452,3	932,2	5									
Range	61.4 - 5593.5	9.1 - 5303.4	41.1 - 5600										
**Panuveitis**	**AH**	**CSF**	**Serum**	**Neurosyphilis UTD**	**CSF VDRL**	**CSF FTA Abs**	**CSF Protein**	**CSF Lymphocytes**	**CSF CXCL13 > 10 pg/mL**	**CSF CXCL13 > 250 pg/mL**	**HIV**	**CXCL13 CSF:serum**	**CXCL13 AH:serum**
1	5539,09	Insufficient	334,61	No	0	Negative	0,51	Insufficient	Insufficient	Insufficient	Yes	Insufficient	16,6
2	5600	1989,93	1989,93	Yes	0	Positive	0,52	35	Yes	Yes	Yes	1	2,8
3	1022,74	5600	1351,8	Yes	0	Positive	1,05	160	Yes	Yes	Yes	4,1	0,8
4	5600	601,98	646,3	Yes	Not available	Not available	0,61	13	Yes	Yes	Yes	0,9	8,7
5	52,38	68,52	450,26	Yes	2	Positive	0,53	7	Yes	No	Yes	0,2	0,1
6	5600	3503,91	255,6	Yes	8	Positive	1,79	84	Yes	Yes	Yes	13,7	21,9
7	5281,65	2318,29	359,3	Yes	0	Positive	0,82	67	Yes	Yes	Yes	6,5	14,7
8	337,23	11,6	346,35	No	Discontinued	Positive	0,21	1	Yes	No	Yes	0,03	0,9
Mean	3629,1	2013,5	716,8	6									
Range	52.38 - 5600	11.6 - 5600	255.6 - 1989.9										
**Optic neuropathy**	**AH**	**CSF**	**Serum**	**Neurosyphilis UTD**	**CSF VDRL**	**CSF FTA Abs**	**CSF Protein**	**CSF Lymphocytes**	**CSF CXCL13 > 10 pg/mL**	**CSF CXCL13 > 250 pg/mL**	**HIV**	**CXCL13 CSF:serum**	**CXCL13 AH:serum**
1	9,12	243,17	48,67	Yes	2	Positive	0,77	27	Yes	No	No	4,9	0,2
2	9,12	5600	9,12	No	0	Negative	0,95	44	Yes	Yes	No	614	1
3	18,56	16,3	269,28	Yes	0	Positive	0,27	6	Yes	No	No	0,06	0,07
Mean	12,3	1953,2	109	2									
Range	9.1 - 18.6	16.3 - 5600	9.1 - 269.3										
**Intermediate uveitis**	**AH**	**CSF**	**Serum**	**Neurosyphilis UTD**	**CSF VDRL**	**CSF FTA Abs**	**CSF Protein**	**CSF Lymphocytes**	**CSF CXCL13 > 10 pg/mL**	**CSF CXCL13 > 250 pg/mL**	**HIV**	**CXCL13 CSF:serum**	**CXCL13 AH:serum**
1	5600	5600	919,5	Yes	0	Positive	0,04	21	Yes	Yes	Yes	6,1	6,1

(*UTD – patients with CSF findings consistent with neurosyphilis according to UpToDate guidelines for diagnosing neurosyphilis).

Analysis of the CXCL13 AH to serum ratio showed 14 patients to have a ratio of more than 1 (71.4%). Not one of the three patients with optic neuropathy had a ratio of 1 or more. An increased ratio was evident in 89% of patients with anterior uveitis, 62% with panuveitis and in the single patient with intermediate uveitis.

Of the 21 available samples, 17 (81%) had AH CXCL13 levels of more than 250 pg/mL, with the lowest levels in the patients with optic neuritis. Except for 2 of the patients with optic neuropathy, all patients had an AH CXCL13 level of more than 10 pg/mL.

## Discussion

Marra et al. found that, compared to patients with uncomplicated syphilis, patients with neurosyphilis who also tested positive for HIV had increased CSF and serum concentrations of CXCL13 despite CSF pleocytosis (18). They found that using a CSF CXCL13 cut-off value of 10 pg/ml had a significantly higher sensitivity (sensitivity 90%; specificity 37%) for diagnosing symptomatic neurosyphilis than using VDRL tests. Using a CXCL13 cut-off value of 250 pg/ml (sensitivity 41%; and specificity 79%) or a CSF to serum ratio of >1 (sensitivity 58%; specificity 84%) was comparable to CSF VDRL testing (18). The CXCL13 concentrations in the CSF decline after treatment for neurosyphilis is initiated and can be used as an indicator for the response to treatment (18, 19, 21, 26).

Mothapo et al. measured the levels of CXCL13 in the CSF of patients with neurosyphilis and uncomplicated syphilis who tested positive or negative for HIV. They used a positive CSF rapid plasma reagin (RPR) test, and not the clinical symptoms, to diagnose neurosyphilis and found a CXCL13 cut-off value of 76.3 pg/ml to be 50% sensitive and 90% specific for diagnosing neurosyphilis (22). The use of CXCL13 as an adjunctive test for patients who tested HIV-negative has been shown to be valuable (22), with significantly elevated levels of CXCL13 in the CSF compared with the serum, indicating intrathecal CXCL13 production in patients with neurosyphilis (21, 25). Zeng et al. could not identify a cut-off value for serum CXCL13 concentrations to differentiate between neurosyphilis and other infectious (viral and cryptococcal) and non-infections central nervous system (CNS) pathology, but the CSF CXCL13 levels were markedly increased in the neurosyphilis group compared with the other groups (25).

Wang et al. identified 3 potential biomarkers for the diagnosis of neurosyphilis. These were CXCL13, CXCL10, also known as interferon-gamma-induced protein 10 (IP10) and CXCL8, also known as interleukin 8 (IL8). The chemokines CXCL8 and CXCL10 also belong to the CXC chemokine subfamily (19). CXCL10 is induced by IFN-γ and promotes adhesion of T-cells, attracts mononuclear cells and enhances innate immunity. CXCL8 enhances the inflammatory cascade involved in the breakdown of the blood brain barrier. Both CXCL10 and CXCL8 are inflammatory chemokines (19). Wang et al. investigated 36 chemokines in the CSF of patients with neurosyphilis who tested negative for HIV and compared it with that of patients with uncomplicated systemic syphilis. They found that CXCL13, CXCL10 and CXCL8 levels were significantly increased in the neurosyphilis group. The optimal cut-off values were 256.4 pg/ml, 163.1 pg/ml and 48.1 pg/ml for CXCL13, CXCL10 and CXCL8, respectively. Above these values CSF CXCL13 was 85.4% sensitive and 89.1% specific for diagnosing neurosyphilis, and CXCL10 and CXCL8 were both 79% sensitive, with a specificity of 90.1% and 91.1%, respectively (19). The sensitivity and specificity of the CSF to serum ratio of CXCL13, CXCL10 and CXCL8 correlated with these values (19). In addition, the decrease in the CSF levels of CXCL10 and CXCL8 was consistent with the decrease of CXCL13 levels after treatment for neurosyphilis was initiated (19).

In our study, the biomarkers CXCL13, CXCL10 and CXCL8 show a trend to be consistently elevated in the AH and CSF of patients with ocular syphilis, with no significant differences between these two compartments. This appears to confirm the relationship between ocular syphilis and neurosyphilis, showing that the intraocular fluid mirrors the intrathecal immunological biomarker milieu in ocular syphilis. The wide variability in the range of the levels and the small study group can exaggerate the mean values, and further studies are needed to confirm these findings.

There were marked differences in the mean concentrations of the 3 biomarkers in the AH and serum (CXCL13 p=0.01, CXCL10 p<0.01 and CXCL8 p<0.01), with higher levels in the AH than the serum. This may indicate that the eye does not merely reflect the serum chemokines that move across the blood-ocular barrier but rather that it is a site of inflammation that produces chemokines.

When comparing patients with and without neurosyphilis, the mean biomarker values, especially CXCL13, did not show significant differences.

The following statistically significant findings became apparent when we compared the group diagnosed as having neurosyphilis with the group diagnosed as not having neurosyphilis: The mean CXCL13 concentration was higher in the AH than the serum of participants with neurosyphilis (p=0.02). The mean CXCL10 concentrations were higher in the AH (p<0.01) and CSF (p=0.01) than in the serum of patients with neurosyphilis. The mean AH concentrations of CXCL8 in participants who were not diagnosed with neurosyphilis were higher than the serum concentrations in participants both with and without neurosyphilis.

In patients with ocular syphilis and neurosyphilis the AH/serum ratios for CXCL13, CXCL10 and CXCL8 were consistently higher than those in patients with ocular syphilis but no neurosyphilis. Further research is required to determine whether or not these ratios could potentially become useful in future in deciding whether someone with ocular syphilis may or may not have concurrent neurosyphilis. Using CXCL13 as an example, the overall AH/serum ratio is 3.33 whereas that for patients without concurrent neurosyphilis is only 2.82 and for patients with concurrent neurosyphilis it is 3.78. Similarly, the overall CSF/serum ratio for CXCL10 is 52.45 where for patients without neurosyphilis it is 22.72 and for those with neurosyphilis it is 73.51. Higher values of the ratio therefore appear to favour a diagnosis of neurosyphilis and lower values the opposite.

Patients with HIV showed elevated CXCL13, CXCL10 and CXCL8 levels when compared to patients without HIV. The mean CXCL10 levels of patients with HIV and without HIV were higher in the AH (p<0.01 and p=0.02) and CSF (p=0.01 and p<0.01) than in the serum. In patients with and without HIV the mean AH level were markedly elevated compared to the serum of HIV positive (p<0.01 and p=0.01) and HIV negative (p=0.02 and p=0.04) patients.

The same trend occurred when dividing the HIV-positive and HIV-negative groups into patients with and without neurosyphilis. The HIV-positive group had higher levels of CXCL13, CXCL10 and CXCL8, but this was not statistically influenced by the neurosyphilis status in these patients with ocular syphilis. The significance of this finding is limited by the small number of patients included in this study.

Using a CSF CXCL13 value of more than 250 pg/mL independently to make the diagnosis of neurosyphilis, would have resulted in 54% being classified as having neurosyphilis, which correlates with the 56.5% of the study sample who have been diagnosed with neurosyphilis. If a CSF CXCL13 level of more than 250 pg/mL was added as an additional criterion to the current UTD guidelines for diagnosing neurosyphilis, another four patients would have been classified as having neurosyphilis. The addition of CXCL13 measurements in the routine testing of our patients would, therefore, have made a difference in the classification of our patients to have neurosyphilis or not. Eighty-one per cent of the patients had AH CXCL13 levels of more than 250 pg/mL

The group of patients with optic neuropathy had the lowest level of AH CXCL13, which confirms the clinical diagnosis of an optic neuritis without intraocular inflammation (uveitis). The CSF results of one patient was inconsistent, with a negative FTA Abs for neurosyphilis, but with the other CSF tests indicating neurosyphilis (CSF CXCL13 level of 5600 pg/mL, 44 lymphocytes, 0.95 g/L protein, CXCL13 CSF to serum ratio of 614, serum RPR of 32). This raises the question whether this patient had a false negative CSF FTA Abs.

With the advances made in recent years regarding the diagnostic capabilities in ascertaining the causes of uveitis, we are moving away from idiopathic uveitis, with more patients receiving targeted treatment for an identified cause. We now propose that the diagnostic certainty for ocular syphilis can also be improved by analysing the AH as an adjunct to the serological testing. This can lead to better outcomes with targeted treatment, especially for patients with HIV, who can have several other causes for infectious uveitis and who also have a higher risk for systemic syphilis.

Although further controlled trials for groups with and without inflammation need to be performed to validate the finding of the biomarker levels in the AH, the above results have a twofold significance. Firstly, it shows that the chemokines measured in the AH of patients with ocular syphilis correlate to the values previously found in the CSF of patients with neurosyphilis, and that patients with ocular syphilis have elevated CSF levels of CXCL13, CXCL10 and CXCL8, as expressed in the mean values (**Figures 1**–**3**). The finding that more than 95% of our patients had a CSF CXCL13 concentration of more than 10 pg/mL also supports the guidelines of the CDC that all patients with ocular syphilis should be treated for neurosyphilis. Secondly, adjunctive analysis of the CXCL13, CXCL10 and CXCL8 levels in the AH of patients with uveitis can be of value in the diagnosis and treatment of uveitis, either as part of a routine workup and assessment of the response to treatment or to confirm or refute a diagnosis of ocular syphilis.

To the best of our knowledge, this is the first study that examined the chemokines CXCL13, CXCL10 and CXCL8 in the AH, CSF, and serum of patients with ocular syphilis. We suggest that the AH can be sampled in addition to testing the serum to confirm the diagnosis of ocular syphilis. This is a novel diagnostic approach for ocular syphilis, and further studies need to be performed to confirm the results and to establish the biomarker levels in normal control groups and patients with other uveitis aetiologies.

Our findings also show that the analysis of the chemokines CXCL13, CXCL10 and CXCL8 in the CSF can indicate the occurrence of neuroinflammation in patients who do not meet the criteria for being diagnosed with neurosyphilis. The addition of CXCL13 analysis in the routine testing of our patients would have resulted in 4 more patients being diagnosed with neurosyphilis. This supports the current guidelines by the CDC that all patients with ocular syphilis need to be treated with the neurosyphilis regime of 10–14 days of intravenous penicillin G.

## Conclusion

CXCL13, CXCL10 and CXCL8 showed AH levels equivalent to previously reported levels in the CSF of patients with neurosyphilis and can potentially be an adjunct in the diagnosis and management of ocular syphilis. Patients with ocular syphilis diagnosed as not having neurosyphilis with conventional CSF testing showed features of neurosyphilis with analysis of the CSF chemokines. This supports the CDC guidelines that all patients with ocular syphilis should be treated with the neurosyphilis regime.

## Data Availability Statement

The original contributions presented in the study are included in the article/supplementary material. Further inquiries can be directed to the corresponding author.

## Ethics Statement

The studies involving human participants were reviewed and approved by the Health Research Ethics Committee of the University of Stellenbosch (N13/10/146). The patients/participants provided their written informed consent to participate in this study.

## Author Contributions

LM was responsible for patient consent, collection of samples and clinical information, literature review and preparation of the article. DS was responsible for conceptualisation of the research and editing of the final article. GW, CS and NC were responsible for analysing the samples and editing the final manuscript. MK was responsible for statistical analysis and interpretation as well as editing the final manuscript. All authors contributed to the article and approved the submitted version.

## Funding

This research was funded exclusively by internal research funds of the Division of Ophthalmology, Faculty of Medicine and Health Sciences, Stellenbosch University, Cape Town, South Africa.

## Conflict of Interest

The authors declare that the research was conducted in the absence of any commercial or financial relationships that could be construed as a potential conflict of interest.

## Publisher’s Note

All claims expressed in this article are solely those of the authors and do not necessarily represent those of their affiliated organizations, or those of the publisher, the editors and the reviewers. Any product that may be evaluated in this article, or claim that may be made by its manufacturer, is not guaranteed or endorsed by the publisher.

## References

[1] Ghanem K Ram S Rice P. The Modern Epidemic of Syphilis. NEJM (2020) 382(9):845–54. doi: 10.1056/NEJMra1901593 32101666

[2] Bowen V Davis D Fleming M Grey J Grier L Harvey A.Sexually Transmitted Disease Surveillance, 2019. In: Centers for Disease Control and Prevention. Available at: https://www.cdc.gov/std/statistics/2019/default.htm.

[3] Zhang T Zhu Y Xu G. Clinical Features and Treatments of Syphilitic Uveitis: A Systematic Review and Meta-Analysis. J Ophthalmol (2017) 2017:1–15. doi: 10.1155/2017/6594849 PMC5511639 28751982

[4] Doris J Saha K Jones N Sukthanka A. Ocular Syphilis: The New Epidemic. Eye (2005) 20(6):703–5. doi: 10.1038/sj.eye.6701954 15933744

[5] Hughes E Guzowski M Simunovic M Hunyor A McCluskey P. Syphilitic Retinitis and Uveitis in HIV-Positive Adults. Clin Exp Ophthalmol (2010) 38(9):851–6. doi: 10.1111/j.1442-9071.2010.02383.x 20659137

[6] Eslami M Noureddin G Pakzad-Vaezi K Warner S Grennan T. Resurgence of Ocular Syphilis in British Columbia Between 2013–2016: A Retrospective Chart Review. Can J Ophthalmol (2020) 55(2):179–84. doi: 10.1016/j.jcjo.2019.11.002 31889521

[7] Oliver G Stathis R Furtado J Arantes T McCluskey P Matthews J. Current Ophthalmology Practice Patterns for Syphilitic Uveitis. Br J Ophthalmol (2019) 103(11):1645–9. doi: 10.1136/bjophthalmol-2018-313207 31021330

[8] Schulz D Orr S Johnstone Devlin M Sheidow T Bursztyn L. The Many Faces of Ocular Syphilis: Case-Based Update on Recognition, Diagnosis, and Treatment. Can J Ophthalmol (2021) 56(5):283–93. doi: 10.1016/j.jcjo.2021.01.006 33549544

[9] Rautenbach W Steffen J Smit D Lecuona K Esterhuizen T. Patterns of Uveitis at Two University-Based Referral Centres in Cape Town, South Africa. Ocul Immunol Inflamm (2017) 27(6):868–74. doi: 10.1080/09273948.2017.1391954 29120678

[10] Smit D Esterhuizen T Meyer D de Boer J de Groot-Mijnes J. The Etiology of Intraocular Inflammation in HIV Positive and HIV Negative Adults at a Tertiary Hospital in Cape Town, South Africa. Ocul Immunol Inflamm (2018) 27(2):203–10. doi: 10.1080/09273948.2018.1476555 29847196

[11] Schaftenaar E Meenken C Baarsma G Khosa N Luijendijk A McIntyre J. Uveitis Is Predominantly of Infectious Origin in a High HIV and TB Prevalence Setting in Rural South Africa. Br J Ophthalmol (2016) 100(10):1312–6. doi: 10.1136/bjophthalmol-2016-308645 27307174

[12] Aldave A King J Cunningham E. Ocular Syphilis. Curr Opin Ophthalmol (2001) 12(6):433–41. doi: 10.1097/00055735-200112000-00008 11734683

[13] Smit D De Graaf M Meyer D de Groot-Mijnes J. Immunoblot and Polymerase Chain Reaction to Diagnose Ocular Syphilis and Neurosyphilis in HIV-Positive and HIV-Negative Patients. Ocul Immunol Inflamm (2020) 28(7):1049–55. doi: 10.1080/09273948.2019.1698753 31944129

[14] Workowski K Bachmann L Chan P Johnston C Muzny C Park I. STI Treatment Guidelines. In: Centers for Disease Control and Prevention. Available at: https://www.cdc.gov/mmwr/volumes/70/rr/rr7004a1.htm.

[15] Troutbeck R Chhabra R Jones N. Polymerase Chain Reaction Testing of Vitreous in Atypical Ocular Syphilis. Ocul Immunol Inflamm (2013) 21(3):227–30. doi: 10.3109/09273948.2013.770887 23617827

[16] Cornut P Sobas C Perard L De Bats F Salord H Manificat H. Detection of *Treponema Pallidum* in Aqueous Humor by Real-Time Polymerase Chain Reaction. Ocul Immunol Inflamm (2011) 19(2):127–8. doi: 10.3109/09273948.2010.531175 21428753

[17] Heymans R van der Helm J De Vries H Fennema H Coutinho R Bruisten S. Clinical Value of Treponema Pallidum Real-Time PCR for Diagnosis of Syphilis. J Clin Microbiol (2010) 48(2). doi: 10.1128/JCM.00720-09 PMC2815629 20007388

[18] Marra C Tantalo L Sahi S Maxwell C Lukehart S. CXCL13 as a Cerebrospinal Fluid Marker for Neurosyphilis in HIV-Infected Patients With Syphilis. Sex Transm Dis (2010) 37(5):283–7. doi: 10.1097/OLQ.0b013e3181d877a1 PMC2884158 20393380

[19] Wang C Wu K Yu Q Zhang S Gao Z Liu Y. CXCL13, CXCL10 and CXCL8 as Potential Biomarkers for the Diagnosis of Neurosyphilis Patients. Sci Rep (2016). doi: 10.1038/srep33569 PMC5030708 27650493

[20] Marra C Gonzalez-Scarano F. Neurosyphilis: Diagnostic Algorithims. Available at: https://www.uptodate.com.

[21] Yan Y Wang J Qu B Zhang Y Wei Y Liu H. CXCL13 and TH1/Th2 Cytokines in the Serum and Cerebrospinal Fluid of Neurosyphilis Patients. Medicine (2017) 96(47):e8850. doi: 10.1097/MD.0000000000008850 29381995 PMC5708994

[22] Mothapo K Verbeek M van der Velden L Ang C Koopmans P van der Ven A. Has CXCL13 an Added Value in Diagnosis of Neurosyphilis? J Clin Microbiol (2015) 53(5):1693–6. doi: 10.1128/JCM.02917-14 PMC4400737 25788544

[23] Kowarik M Cepok S Sellner J Grummel V Weber M Korn T. CXCL13 Is the Major Determinant for B Cell Recruitment to the CSF During Neuroinflammation. J Neuroinflam (2012) 9(1). doi: 10.1186/1742-2094-9-93 PMC3418196 22591862

[24] Hu R Lu C Lu S Hu Y Ma H Lai W. Value of CXCL13 in Diagnosing Asymptomatic Neurosyphilis in HIV-Infected Patients. Int J STD AIDS (2015) 27(2):141–6. doi: 10.1177/0956462415577229 25769888

[25] Zeng Y Lin Y Zhang N Zou C Zhang H Peng F. CXCL13 Chemokine as a Promising Biomarker to Diagnose Neurosyphilis in HIV-Negative Patients. SpringerPlus (2016) 5(1). doi: 10.1186/s40064-016-2462-4 PMC4909691 27376011

[26] Dersch R Hottenrott T Senel M Lehmensiek V Tumani H Rauer S. The Chemokine CXCL13 is Elevated in the Cerebrospinal Fluid of Patients With Neurosyphilis. Fluids Barriers CNS (2015) 12(1). doi: 10.1186/s12987-015-0008-8 PMC4489031 25975424

[27] Bremell D Mattsson N Edsbagge M Blennow K Andreasson U Wikkelsö C. Cerebrospinal Fluid CXCL13 in Lyme Neuroborreliosis and Asymptomatic HIV Infection. BMC Neurol (2013) 13(1). doi: 10.1186/1471-2377-13-2 PMC3546953 23294475

